# Protocol for a systematic review: Interventions to improve mathematics achievement in primary school‐aged children: a systematic review

**DOI:** 10.1002/CL2.215

**Published:** 2018-03-09

**Authors:** Victoria Simms, Camilla Gilmore, Seaneen Sloan, Clare McKeaveney

## BACKGROUND

### The problem, condition or issue

The role of mathematics has become even more important an ever before (Xing, 2013) as everyday life requires some form of mathematical understanding and decision‐making. Yet low achievement in mathematics affects a large proportion of the general population. Dowker (2009) estimated 15‐20% of the general population have some form of mathematical learning difficulty. In fact, it is estimated within the UK, 15‐17 million adults have poor numeracy skills (Every Child a Chance Trust, 2009; Skills for life, 2012). Research has focused on interventions for those with a suspected (i.e. low achievement) or diagnosed mathematics learning disabilities (e.g. Dyscalculia). Yet, there are no consistent standards by which to judge the presence of a math learning disability nor is there agreement concerning the precise definition, criteria, or prevalence (Karagiannakis, Baccaglini‐Frank &Papadatos, 2014). A recent report suggests that approximately 5% of the UK population meet the diagnostic criteria for Dyscalculia (Department of Work & Pensions, 2017)but this only accounts for a small percentage of the total population (Dowker&Sigley, 2010; Mazzocco, 2015).

A greater number of students struggle with low math performance without a disability diagnosis (Nelson & Powell, 2017). Many children struggle to meet expected levels in mathematics by the end of primary school (DE, 2015). Gross (2007) reported that 21% of 11‐year‐olds leave primary school without reaching the mathematical level expected, and 5% do not attain the mathematical level expected of a 7‐year‐old. Most alarming are the negative outcomes associated with low numeracy, such as significantly higher risk of unemployment, lower wages, mental health problems, physical illness, arrest and incarceration (Gross, Hudson, and Price, 2009). Moreover, the societal cost of low numeracy is considered to include billions of pounds worth of lost revenue in taxes and welfare provision (Every Child a Chance Trust, 2009; OECD 2010). Focusing mathematical research on such larger homogenous samples affected by low numeracy is therefore necessary to provide greater understanding of how to increase mathematical learning across a wider population.

Increased investment into mathematical research has led to a plethora of studies identifying a wide breadth of domains involved in mathematical difficulty as well as countless interventions. Deficits include attitudes, motivation, language ability and IQ, in addition to social and educational factors. Studies have identified executive functioning skills such as holding and manipulating information in mind, the ability to suppress distracting information and unwanted responses and shifting, and the ability to flexibly switch attention between different tasks as also implicated in mathematics achievement (Cragg& Gilmore, 2014; Cragg, Keeble, Richardson, Roome& Gilmore, 2017). Additional factors that could cause low achievement in mathematics include anxiety, stress and low self‐esteem (Chinn & Ashcroft, 2007). To the same extent that not all students learn in the sameway, not all children fail to learn mathematics in the same way (Wardrop, 2014). Math learning draws on several cognitive mechanisms and memory systems highlightinga highly complex learning process. Difficultiescan arise from a wide‐range of areas emphasising why establishing a reliable diagnosis of dyscalculia or math difficultly can be challenging (Kormos, 2012).

Learning mathematics in the early years (e.g. primary education) determines how much success children have later in math and as well as other areas such as reading (Sarama and Clements, 2009). The traditional approach to mathematics involves the explicit teaching of procedures and algorithms followed by fluency through repeated practice, known as ‘direct instruction’. However, Sweller, Clark and Kirschner (2010) argue, “instructional guidance in mathematics leads to minimal learning” (p.1304). Research on math instruction approaches have found that more creative forms of instruction can be more effective than traditional methods. Another approach involves consolidating math learning using rich real‐life situations known as ‘problem‐based’ or ‘inquiry‐based’ instruction (Hattie et al., 2016) which can increase metacognitive skills that are critical to future success in mathematics (Marschark et al., 2002).

Recent research has been dedicated to developing effective interventions to improve mathematical learning (DE, 2012). However, this has not resulted in a clearer understanding what does and does not improve mathematical learning for children with poor numeracy skills. Recent reports have called for an increase in systematic investigations of education interventions to provide an evidence‐base to inform decisions about educational changes (DE, 2013). Therefore, a broad review of effective interventions to improve mathematical learning for children is timely due to the rapid development of new interventions. An evidence base that includes a broad, rather than narrow, review of intervention literature may benefit every teacher and all children in the classroom.

### Intervention

Systematically reviewing evidence on the effectiveness of a range of mainstream student‐directed classroom based interventions will allow teachers and policy makers to access reliable and validated research to make informed choices about appropriate teaching practices and interventions. The included trials will be classroom‐based mathematics interventions directed at primary school‐aged pupils, such as those applied at the whole‐class level and one‐to‐one support, delivered by trained teaching professionals as well as through other mediums such as peer tutoring. The term ‘intervention’ is defined as a deviation from standard teaching practice. Trials aimed at parents or requiring parental involvement will not be included as this is beyond the scope of the review. Unlike previous reviews, we will focus on interventions that did not select participants based on suspected or diagnosed mathematical difficulties, i.e. mathematics achievement below the 25th percentile on standardised mathematical tests. Therefore, addressing the need to identify practices that may have the most benefit for large groups of children in the classroom across the achievement spectrum (e.g. those with low or average mathematical achievement). Eligible study designs in this study will focus on randomised controlled designs (RCT). It is important to note that RCTs are not necessarily the most effective educational research method however; they do provide the strongest evidence of effectiveness (Akobeng, 2005).

### How the intervention might work

Several general cognitive and mathematics‐specific skills have been identified as being predictive of mathematical achievement in primary school aged children such as working memory (Raghubar, Barnes & Hecht, 2010), inhibitory control (Cragg& Gilmore, 2014), counting skills (Cowan, Donlan, Shepard et al., 2011) and flexible strategy use (Geary & Brown, 1991). Attitudes and anxious thoughts directed towards mathematics have also been linked to mathematical performance (Maloney, Schaffer &Beilock, 2013). The identification of these skills has directly informed the development of experimental interventions that range from one‐to‐one computerised training to whole‐class pedagogy approaches. Individually administered training has been developed to improve specific mathematical skills for all students such as number sense (Wilson, Dehaene, Dubois & Fayol, 2009) and basic addition skills (Siegler&Ramani, 2008). It has also been proposed that computerised training of working memory may lead to gains in mathematical learning, although evidence for this is mixed (Melby‐Lervag& Hulme, 2013). Classroom‐based educational interventions such as explicit delivery of heuristics, encouraging children to reflect and verbalise their strategy use and explicit instruction have been established to be effective in improving mathematical outcomes (Gersten et al., 2009). Tentatively, the use of concrete manipulatives, such as cuisenaire rods or Numicon^TM^, have been associated with improvements in mathematical learning (Dowker, 2009). Expressive writing interventions that aim to reframe anxious thoughts about mathematics have been observed to improve mathematical performance on a classroom level (Ramirez &Beilock, 2011). In addition, peer tutoring, in which children guide one‐another's learning and provide feedback, has also been observed to assist in mathematical outcomes (Thurston, Tymms, Merrell &Conlin, 2014).

### Why it is important to do the review

Existing research has been unable tobetter identify the key features of effective interventions within randomised controlled designs, due to the inherent contradictions within educational research. This includes:

#### i. A lack of randomised controlled studies

To the authors’ knowledge, there are no systematic reviews specifically exploring the outcomes of interventions focused on improving mathematical learning assessed via randomised controlled trials. Generally, randomised controlled trials within education are less common compared to other experimental designs due to the time and costs involved as well as the ethical concerns regarding the random allocation of, possibly, beneficial interventions (Sykes, Schneider, Plank & Ford, 2012). However, there has been a gradual movement over time towards the inclusion of randomised controlled trials to gain high quality evidence to help inform decision‐making within education (Tranfield, Denyer& Smart, 2003). Randomised controlled trials provide the most rigorous method of determining cause‐effect (Sibbald, 1998). Systematically reviewing such studies would provide greater validity and reliability regarding the outcomes of these interventions.

#### ii. Largely dyscalculic samples

Within the literature there is a focus on mathematical interventions with specialist populations of children, for example children with Dyscalculia (Gersten et al., 2009; Kroesbergen& Van Luit, 2003; Xin &Jintendra, 1999). Currently, there is an ongoing registered Cochrane review focusing on mathematical interventions to improve mathematical learning in children with Dyscalculia (Furlong et al., 2016), the protocol of which has been recently published. Current theories suggest that children with Dyscalculia struggle with mathematics due to specific cognitive deficits (Geary, 2011) not shared by individuals with general low achievement in mathematics. Consequently, children with Dyscalculia benefit from different types of intervention compared with children in the rest of the achievement spectrum. Accordingly, this highlights the need to systematically review interventions targeted improving mathematical learning across the achievement spectrum.

#### iii. Mixed age groups

Wide age‐ranges of populations (i.e. both primary and secondary levels) are included in published systematic reviews (Cheung &Slavin, 2013; Carbonneau, Marley & Selig, 2012) of mathematical interventions (e.g. children and adolescents). However, it has been suggested that early interventions are the most cost effective and efficient approach to improving children's educational outcomes (Easton & Gee, 2012). Therefore, discrepancies between studies concerning the impact of interventions on learning may be attributed to comparing studies that intervene on young children to those that focus on adolescents. This further emphasises the need for a systematic review that provides a clearly focused criterion (i.e. on primary school sample; implemented in school settings) to allow interpretation and application by the relevant practitioners and policy makers.

#### iv. Narrow reviews of intervention methods

Existing systematic reviews that do include children across the ability spectrum also tend to focus on a specific type of interventions, such as educational technology applications (Cheung and Slavin, 2013) or the use of concrete manipulatives (Carbonneau, Marley and Selig, 2012). In addition, such reviews contain varied methodologies by including a wide range of experimental designs, duration of interventions, sample characteristics and year of publication. This increases the likelihood of several methodological biases and ultimately reduces the quality of findings. In addition, identifying similar contexts and sample characteristics becomes difficult. Ultimately, such narrow reviews may not assist general educational practitioners informed decision making. Therefore, a systematic investigation that allows comparison between intervention types within relevant contextual features (e.g. primary school children in mainstream school) will provide a cohesive evidence‐base, benefiting teachers.

### Products of this systematic review

By addressing these issues, this review will be to provide practitioners and policy makers with reliable and valid contextual information about interventions that improve children's mathematical outcomes. More specifically, this review will enable teaching professionals to make informed, evidence‐based decisions that is substantiated through studies carried out in similar contexts and with similar sample characteristics to their own pupils. The proposed systematic review will focus on primary school aged children, identifying content of interventions and assessing the efficacy of these interventions. If possible, meta‐analysis of effect sizes across studies will provide a clear scientific evidence‐base to inform and support decision‐making by teachers, head‐teachers and policy makers. A key additional tool for dissemination will also be a website and handbook for teachers and policy makers. The “What Works Clearinghouse” (WWC)is a like‐mindedresource that evaluates classroom curricula, programs and materials within educational research. However, unlike the WWC, the product of this review (e.g. website) will involve significant discussion and inputfrom the teaching community. The website will provide a summary of numeracy interventions suitable for primary school children, which will be searchable by key words, cost, implementation process and materials to enable teachers and policy makers to better identify relevant interventions. By providing a dedicated study website and additional tools such as a handbook and training events, this review will address a current gap in available information for educational professionals.

## OBJECTIVES

This systematic review will aim to answer the following questions:

(1) What types of classroom‐based interventions or programmes are used with primary school‐aged children who do not meet the criteria for math disability?
(2) What are the most effective classroom based interventions for improving mathematical learning in primary school‐aged children who do not meet the criteria for math disability?
(3) What are the key characteristics of the most effective interventions for improving mathematical learning primary‐school aged childrenwho do not meet the criteria for math disability (e.g. equipment involved, any associated costs)?

## METHODOLOGY

### Criteria for including and excluding studies

*Types of study designs*

Included:

- Randomised controlled trials (including cluster randomised controlled trials)
- Must include comparison control condition (e.g. no intervention, practice‐as‐usual, waiting list, or active control group)

Excluded:

- Quasi‐randomised designs
- Matched subject or group designs
- Cross‐over designs
- Single‐subject designs
- Correlational designs

*Time and language*

- All eligible studies will be published from January 2000, this will ensure that the materials included are relevant in terms of curriculum context, to the time of literature search conclusion (approximately August, 2017). Full texts must be available in English.

*Types of participants*

Included:

- Participants are children aged 4‐11 years’ old
- Attending mainstream primary/elementary‐level schools

Note: If a study only mentions Grade level, rather than age, the following criteria will be applied. Students between Kindergarten up to Grade 6 will be included *only if* students are situated within a primary/elementary school environment (e.g. middle school Grade 6 will be excluded despite including 11‐12 year olds).

Excluded:

- Samples selected due to suspected or diagnosed mathematical difficulties (e.g. mathematics achievement below the 25th percentile or less on standardized mathematical tests)
- Samples attending special education establishment
- Samples with other diagnosed learning difficulties or developmental disorders

*Types of interventions*

Included:

- Interventions (specific deviations from standard practice)aimed at improving mathematicskills (e.g. speeded recall of arithmetic facts, flexible strategy use)
- Interventions carried out in school settings, not laboratory based studies
- Administrated individually, within small groups or whole‐class based
- Delivery method may include those delivered by teachers, teaching/classroom assistants or other trained professionals
- At least one follow‐up at post‐test is necessary (within one month)

Excluded:

- Interventions aimed at children screened or suspected of Dyscalculia
- Interventions aimed at parents and requiring parent participation

*Types of outcome measures*

The primary outcome is mathematics ability, as measured by:

- Curriculum‐based outcome measures (e.g. Key Stage assessments)
- Standardised tests (e.g. Wechsler Individual Achievement Test Numerical Operations or Mathematical Reasoning)
- Cognitive experimental measures of specific mathematics skills (e.g. speeded recall of arithmetic facts, flexible strategy use)

Secondary outcomes will include attitudinal or affective constructs, e.g. attitude towards mathematics; mathematical anxiety levels; mathematical self‐efficacy; confidence in mathematics skills or enjoyment of the subject. In addition, if possible identifiable costs (e.g. unit costs or costs per student, technology costs) will be extracted and summarised.

*Duration of follow‐up*

- Immediate post‐test results (up to 30 days post intervention)
- If longer term follow‐up periods vary greatly (e.g. after 6 months, after 12 months) then similar follow‐up periods may be grouped

Note: The primary analysis will be based on immediate post‐test data. If sufficient data are available, separate meta analyses will be conducted for outcomes measured at longer follow up periods (e.g. up to 6 months post‐intervention and up to 12 months post‐intervention).

*Types of settings*

- Interventions (specific deviations from standard practice) directed towards students in mainstream classroom settings

### Search strategy

Electronic search

Eligible studies will have been published since January 2000, in English. To identify all eligible studies, an extensive search will be conducted using the following databases:

- British Educational Index
- Education Abstracts
- Academic Search Complete
- MEDLINE
- ERIC
- PsycINFO
- Social Sciences Citation Index
- International Bibliography of the Social Sciences
- Applied Social Sciences Index and Abstracts
- Cochrane Central Register of Controlled Trials
- Evidence for Policy Practice Information and Coordinating Centre (EPPI‐Centre)

Unpublished and grey literature:

- ProQuest Dissertations and Thesis
- Dissertation Abstracts International
- Conference Proceedings Citation Index
- Websites of charitable and funding organisations (Nuffield Foundation, National

Numeracy Trust, the Education Endowment Foundation and National Science Foundation (USA))

- Government departments (e.g. Department of Education)
- World Health Organisation trials website and clinicaltrials.gov)
- Google and Google Scholar (e.g. first 150 hits recorded, exact URL and date of access recorded)
- OpenGrey

As suggested in the C2 Literature Search guide (Hammerstrøm, Wade, &Jørgensen, 2010), journal hand searches will be conducted where many included studies have been found (e.g. British Journal of Educational Psychology). Reference lists of all identified articles or review articles will be checked. Prominent authors in the field will also be contacted.

### Search terms

**Table 1:** Search terms

Population	((Primary OR Elementary OR Kindergarten* OR “Grade 1” OR “Grade 2”, “Grade 3”, “Grade 4”, “Grade 5”) AND (school* OR educat* OR class* OR teach* OR learn* OR instruct* OR train* OR program*))
Intervention	(Math* OR “Number Sense” OR Numer* OR Arithmetic* OR counting OR addition OR subtraction OR multiplication OR division OR Adding OR Geometry OR fractions OR algebra OR “place value”)
Outcomes	(Achieve* OR “Standardi* Test” OR Anxiety OR Attitud* OR “Self‐Efficacy” OR Confidence OR Enjoyment)
Methods	(Trial OR RCT OR Random* OR “Control Group” OR “Post Test” OR experimental)

One trained research assistant (CM) will conduct the initial search following the search strategies above. Training has involved information retrieval workshops and specialist mentorship with Cochrane Ireland and the Campbell Collaboration. Titles and abstracts will be screened independently by two of the review team and coded (on the basis of inclusion/exclusion criteria) in EPPI‐Reviewer. One author (CM) will undertake coding of all identified studies, with second coding of each study divided equally between the other three reviewers (VS, CG and SS). Disagreements between the review team (e.g. CM and VS) will be resolved by a different review team member (e.g. either CG or SS) and consensus will be achieved. Full texts of potentially eligible studies will be located and again screened independently by two of the review team. Again, disagreements will be resolved through discussion and consensus as a team. Reasons for excluding studies will be clearly documented and reported.

#### Description of methods used in primary research

Studies in the systematic review will consist of randomised controlled trials (RCT). This review will use include both pre‐test and post‐test measurements. Comparison group conditions will involve treatment as usual, other appropriate interventions, or no interventions. All identified eligible studies will be summarised in a ‘Summary of Findings Table’ in the completed review.

The following two studies provides example eligibility criteria for the proposed review:

**Barner and colleagues (2016)** conducted a randomised controlled trial to assess the impact of a mental abacus technique on students’ mathematical abilities within a low socioeconomic status school in India over three years. Children aged 5 to 7 years old were randomly assigned to an intervention group to use the mental abacus technique or control group of standard curriculum maths. The intervention included three hours per week (2 x 90 min sessions) of instruction in the use of the physical and mental abacus by an experience mental abacus teacher. The mathematics outcome measured four mathematical tasks; using the Math Fluency subset of the Wechsler Individual Achievement Test (WIAT‐III); the Calculation subtest of the Woodcock Johnson Tests of Achievement (WJ‐IIIC); and two specifically designed tests to target arithmetic and place value knowledge. The study also assessed cognitive, academic and attitudinal outcomes but no significant changes were reported. However, the mental abacus technique led to **significant gains** in the Calculation subtest (Cohen's d=0.60;CI:0.30‐0.89), arithmetic (Cohen's d=0.24;CI:‐0.05‐0.52) and place value (Cohen's d=0.28;CI: 0.00‐0.57) knowledge suggesting use of a mental abacus is an effective tool for improving math performance.

**McNeil**, Fyfe and Dunwiddie (2015) conducted a randomised controlled trial on the effectiveness of a modified presentation of traditional arithmetic practice workbooks. Children aged 7‐8 years old were recruited from three schools, two serving children from low socioeconomic backgrounds. To assess understanding of math equivalence a modified workbook (e.g. _ = 4+3) or a control workbook with a traditional layout (e.g. 4+3= _) was presented to children. In addition, the study assessed ‘computational fluency’ using the Math Computation section of the Level 8 of the Iowa Test of Basic skills along with a timed paper and pencil addition test designed by Geary et al. (1996) (e.g. number of single digit addition facts answered correctly in one minute). **No significant changes** in computational fluency were noted but the intervention group showed better understanding of mathematical equivalence compared to the control group, suggesting that even minor modifications to the presentation of math problems can lead to a greater understanding of mathematical concepts.

#### Criteria for determination of independent findings

Non‐independence of findings can occur when multiple measures of the same outcome are reported in a single study. Mathematics ability is usually directly assessed, meaning the issue of multiple informants of the same outcome is unlikely to be a major issue for our primary outcome, however where this is identified, we will calculate an average weighted effect size within each study for each outcome. Where the same outcome is measured at multiple time‐points (e.g. immediate post‐test, 6 month follow up, 12 month follow up), analysis will focus on the time‐point closest to the end of the intervention period. Where multiple reports of the same study are identified through scrutinising author names, sample description and study time frame, data will only be extracted once from the most complete and detailed report.

#### Details of study coding categories

Included studies will be coded in Excel by CM. The type of information that will be coded includes: study design (including unit of allocation and description of trial arms), geographic location, relevant sample information (e.g. age, sex, ethnicity, socio‐economic status), initial sample size and attrition, intervention type, delivery mode (e.g. teacher, trained classroom assistant, etc) and personnel, frequency (e.g. daily, weekly, bi‐monthly, etc) and duration (e.g. one off session, one year, etc) implementation fidelity, outcomes and measures used, intervention effects, and any reported data on intervention costs. In addition, any available contextual information (e.g. school population) relating to study setting/sample characteristics will be extracted from the included articles. A coding sheet is attached as an appendix.

Included studies will be assessed for risk of bias by two reviewers, using the Cochrane Collaboration's Risk of Bias tool (Higgins & Green, 2011). Studies will be categorised as having ‘low’, ‘high’ or ‘unclear’ risk of bias based on the following features:

*Random sequence generation*: Studies will be categorised as low risk if the method used to generate the allocation sequence is described in sufficient detail and able to produce comparable groups.

*Allocation concealment*: Studies will be categorised as low risk if the allocation sequence was adequately concealed from study personnel or participants, such that it would not be possible to predict which group participants would be allocated to.

*Blinding of participants and personnel*: Lack of blinding can lead to different expectations for individual's performance, thus biasing trial results. Studies will be coded as low risk if participants (teachers and pupils) and trial personnel are blind to allocation status.

*Blinding of outcomes assessors*: Studies will be coded for outcome assessors and whether assessors are blind to allocation status. Studies will be coded as low risk of bias if outcomes are assessed by independent fieldworkers or invigilators who have no knowledge of group allocation.

*Incomplete outcome data*: The extent of missing outcome data by group allocation will be extracted to assess for systematic differences between intervention and control groups in terms of measurement attrition and the reasons for missing data. Studies with no attrition, low attrition (<20%), or no evidence of differential attrition will be coded as having low risk of bias. Use of intention to treat (ITT) analysis and methods of account for missing data (e.g. using multiple imputation), will be recorded.

*Selective outcome reporting*: Reporting biases will be assessed, that is whether there is discord between the outcomes measured and those reported (which may be detailed in the methods section of trial reports, or in separate trial protocols). Studies at low risk of bias will either have published a protocol detailing pre‐specified outcomes of interest or clearly show through the methods and results sections of publications that all pre‐specified outcomes are reported.

#### Statistical procedures and conventions

Included studies will be synthesised via narrative and statistical methods. Meta‐analysis will be conducted if there are sufficient (i.e. over 10) included studies that can be meaningfully grouped together. It is essential that a logical approach is taken when making decisions on combining studies in meta‐analyses. Studies will only be combined if there are a) a reasonable number of studies with shared characteristics and b) those studies share characteristics in terms of the type of intervention and the outcome that the intervention targeted. These characteristics are important as it would not be logical to combine effects of widely different types of interventions or studies that focus on improving very different facets of mathematics.

Meta analysis will be conducted in Comprehensive Meta Analysis (CMA, Borenstein et al., 2005) using random‐effects models. Separate analyses will be conducted for the primary outcome (mathematics ability) and secondary outcomes (attitudinal and affective outcomes). The experimental versus control comparisons will focus on adjusted post‐test means which control for imbalance at pre‐test. If this detail is not available, we will subtract the pre‐test mean effect size from the post‐test mean effect size. The unadjusted pooled standard deviation will be used. For continuous data, we will use mean differences for similar outcome measures (e.g. standardised achievement tests) with a 95% confidence interval. However, it is likely that outcome measures will differ between studies, and where this is the case, we will use the standardised mean difference as the effect size metric based on Hedges’ g which adjusts for bias associated with small samples, which is calculated using the following formula:

$$g=\frac{M_{1}-M_{2}}{SD_{*pooled}}$$

Note: M_1_ – M_2_ represents the difference on an outcome between the control and intervention group that is divided by the pooled weighted standard deviation of the outcome.

Dichotomous outcome data will be analysed by calculating the risk ratio (and its 95% confidence interval) for the occurrence of an event. Risk ratios will be converted to the standardised mean difference (using David Wilson's practical effect size calculator) for the purpose of meta‐analysis.

Meta‐regression will be used to examine the influence of moderating variables on intervention effect size. Moderator analysis will be carried out when a) there are a reasonable number of publications identified that can be meaningfully analysed and b) when the required data is available in the publication. Any subgroup analysis will be clearly labelled as such in the final review.

#### Studies with multiple groups

If a study is identified with two (or more) intervention groups versus one control group, and all interventions are deemed relevant, there are two options as to how these groups should be treated: a) If the intervention groups are similar the two groups should be treated as single group or b) If the intervention groups are not similar the control group sample size will be divided in half and will then be compared to the intervention groups. Thus this study would provide two effect size estimates. This process is carried out to ensure that participants in the control group are not “double counted” (Higgins & Green, 2011). A similar strategy, but in reverse, will be carried out if there is a study with one intervention group and two control groups. In addition, if a paper is identified that contains one relevant and one irrelevant intervention group the data from the relevant group will be analysed, but irrelevant data associated with an irrelevant intervention will be ignored.

#### Unit of analysis issues

It is likely that cluster‐randomised trials will be identified, i.e. studies where participants are allocated as a group rather than individually. If such studies have not been appropriately adjusted for clustering (using, for example, robust standard errors or multi‐level modelling), we will apply standard conversion criteria as outlined in the Cochrane Handbook (Higgins & Green, 2011). We will use ICC estimates pertaining to each individual study, and if these are unavailable, we will identify appropriate external ICCs based on comparable study size and context. The What Works Clearinghouse (US) has recommended an ICC of 0.20 for achievement outcomes, and 0.10 for all other outcomes (WWC, 2017), and the Education Endowment Foundation (2015) estimates ICCs of 0.103 – 0.126 for maths attainment in primary school (EEF, 2015). If it is unclear whether a cluster‐randomised controlled trial has used appropriate controls for clustering, we will contact the study authors for further information.

#### Investigation of heterogeneity

We will assess heterogeneity by comparing the distribution of important factors such as participant demographics, type of intervention and control comparators and outcomes measured across studies. If appropriate, we will synthesise results in meta‐analyses, specifically meta‐regression using the CMA program. Meta‐analyses will use random effects models. If there are sufficient data, we will carry out subgroup analysis to explore the effect of key study attributes, such as a how the intervention was administrated (e.g. by a teacher or educational psychologist, on an individual or group basis), intervention intensity and duration and the impact of pupil characteristics. Intervention intensity will be defined as low (one off session to one month of sessions), medium (> one month of sessions to one year of session) or high (> one year of sessions). High intensity interventions have generally been shown to provide higher gains than low intensity interventions. Early interventions are suggested to be more effective than interventions applied to older children, this again can be assessed in the meta‐analysis. Heterogeneity will be measured and reported through the Q, l^2^ and Tau^2^ statistics in conjunction with visual inspection of forest plots.. Significant heterogeneity will be defined as an l^2^ above 75% (Higgin & Green, 2011). High heterogeneity is expected due to the broad criteria for population inclusion in this systematic review.

#### Sensitivity analysis

Sensitivity analysis will be conducted to determine whether the overall findings of the analysis are impacted by removal of:

- studies with outlier effect sizes;
- unpublished studies;
- studies with missing information (e.g. standard deviations or ICCs) which had to be imputed;
- studies with high risk of bias.

#### Missing data and author queries

If further information is required regarding study data to conduct appropriate analyses or to establish baseline equivalence, the first author of the study will be contacted via email. One reminder email will be sent within one month of first request of information. Should requested data be unavailable, the study will still be reported, but not included in the final meta‐analysis.

#### Treatment of qualitative research

We do not plan to include qualitative research.

### REVIEW AUTHORS

**Lead review author:** The lead author is the person who develops and co‐ordinates the review team, discusses and assigns roles for individual members of the review team, liaises with the editorial base and takes responsibility for the on‐going updates of the review.

**Table:** 

Name:	**Victoria Simms**
Title:	Dr
Affiliation:	Ulster University
Address:	Coleraine, Cromore Road
City, State, Province or County:	Londonderry
Postal Code:	BT57 34s
Country:	Northern Ireland
Phone:	07816056279
Email:	v.simms@ulster.ac.uk
**Co‐author(s):** (There should be at least one co‐author)	
Name:	**Camila Gilmore**
Title:	Dr
Affiliation:	Loughborough University
Address:	Mathematics Education Centre, Loughborough
City, State, Province or County:	Leicestershire
Postal Code:	LE11 3TU
Country:	England
Phone:	+44(0) 1509228218
Email:	c.gilmore@lboro.ac.uk
	
Name:	**Seaneen Sloan**
Title:	Dr
Affiliation:	University College Dublin
Address:	School of Education, Belfield, Dublin 4
City, State, Province or County:	Dublin
Postal Code:	N/A
Country:	Ireland
Phone:	07931257815
Email:	Seaneen.sloan@ucd.ie
Name:	**Clare McKeaveney**
Title:	Miss
Affiliation:	Ulster University
Address:	Coleraine, Cromore Road
City, State, Province or County:	Londonderry
Postal Code:	BT57 34s
Country:	Northern Ireland
Phone:	07736688160
Email:	c.mckeaveney@ulster.ac.uk

### ROLES AND RESPONSIBILITIES

Victoria Simms (Principal investigator) will be responsible for the overall conduct of the review, supervising the work of a full‐time post‐doctoral researcher. Dr Camilla Gilmore (Co‐investigator) is a content expert and will contribute to all aspects of the review. Dr Seaneen Sloan (Co‐investigator) will provide guidance on systematic review methods and will contribute to all aspects of the review. The post‐doctoral researcher (Miss Clare McKeaveney) has attended training on systematic reviews. In addition, we have an expert advisory group with substantial content and methodological expertise (Dr Nuala Livingstone and Dr Sarah Miller).

- Content: Dr Victoria Simms, Dr Camilla Gilmore
- Systematic review methods: Dr Seaneen Sloan, Miss Clare McKeaveney (and advisory panel)
- Statistical analysis: Dr Seaneen Sloan, Miss Clare McKeaveney (and advisory panel)
- Information retrieval: Dr Victoria Simms, Dr Camilla Gilmore, Dr Seaneen Sloan, Miss Clare McKeaveney (and advisory panel)

### FUNDING

Yes, we have received funding from the Nuffield Foundation.

### POTENTIAL CONFLICTS OF INTEREST

No, there are no conflicts of interest.

### PRELIMINARY TIMEFRAME

Plan to submit a draft review: September 2017

### AUTHOR DECLARATION

#### Authors’ responsibilities

By completing this form, you accept responsibility for preparing, maintaining, and updating the review in accordance with Campbell Collaboration policy. The Coordinating Group will provide as much support as possible to assist with the preparation of the review.

A draft protocol must be submitted to the Coordinating Group within one year of title acceptance. If drafts are not submitted before the agreed deadlines, or if we are unable to contact you for an extended period, the Coordinating Group has the right to de‐register the title or transfer the title to alternative authors. The Coordinating Group also has the right to de‐register or transfer the title if it does not meet the standards of the Coordinating Group and/or the Campbell Collaboration.

You accept responsibility for maintaining the review in light of new evidence, comments and criticisms, and other developments, and updating the review every five years, when substantial new evidence becomes available, or, if requested, transferring responsibility for maintaining the review to others as agreed with the Coordinating Group.

#### Publication in the Campbell Library

The support of the Coordinating Group in preparing your review is conditional upon your agreement to publish the protocol, finished review, and subsequent updates in the Campbell Library. The Campbell Collaboration places no restrictions on publication of the findings of a Campbell systematic review in a more abbreviated form as a journal article either before or after the publication of the monograph version in *Campbell Systematic Reviews*. Some journals, however, have restrictions that preclude publication of findings that have been, or will be, reported elsewhere and authors considering publication in such a journal should be aware of possible conflict with publication of the monograph version in *Campbell Systematic Reviews*. Publication in a journal after publication or in press status in *Campbell Systematic Reviews* should

acknowledge the Campbell version and include a citation to it. Note that systematic reviews published in *Campbell Systematic Reviews* and co‐registered with the Cochrane Collaboration may have additional requirements or restrictions for co‐publication. Review authors accept responsibility for meeting any co‐publication requirements.

**Form completed by:**

**Clare McKeaveney**

**Date: 18/09/2017**

**I understand the commitment required to undertake a Campbell review, and agree to publish in the Campbell Library. Signed on behalf of the authors**:

**Table:** 

Item	
Author names	
Year of publication	
Title of publication	
Type of study (RCT/quasi‐experimental etc)	
Rationale for study (paste into cell)	
Study informed by or linked to an exisiting body of empirical and/or theoretical research? (Yes/no)	
Study research question/ hypotheses? (Paste into cell)	
Population of the study	
Age of participants (mean, median, range)	
Gender of participants?	
Was the intervention carried out in a classroom setting? (Yes/no)	
What country was the study carried out in? (Name)	
Was fidelity measured ? (Yes/No)	
Any detail on fidelity? (paste into cell)	
Method of recruitment of participants (e.g. invitation letter, information evening for parents)	
Population description (provide any additional detail on the population studied that is mentioned in the publication)	
Detail any inclusion criteria	
Detail any exclusion criteria	
Total population at start of study (N)	
How many groups were in the study?	
Total number of intervention groups	
Total number of control groups	
What was the basis for group divisions?	
Do the groups differ by any specific detail (e.g. grade, etc)	
Unit of allocation (e.g. individual, class, school, community, etc)	
Description and number of clusters (give details)	
Methods used to analyse the data were explicitly stated (Yes/no/unclear)	
Specifiy the statistical methods used (copy into the cell)	
Rationale for methods of analysis explicitly stated (Yes/no/unclear)	
% of invited individuals agreed to participate	
Total number of participants randomised	
Number of individuals allocated to each group	
For cluster trials, number of clusters and average number of of participants per cluster	
% of participants who completed the study	
% of participants who received intervention % of participants who received control	
Where there any significant baseline imbalances? (Yes/no/none/noted) If yes, what were they?	
Number of withdrawals and exclusions for each intervention group	
Description of intervention (e.g. content, dose, components) (copy content into cell)	
Duration of intervention	
Timing (e.g. frequency, duration of each lesson)	
Delivery (e.g. when during the school day, medium of intervention, intensity of intervention)	
Providers (e.g. teacher, assistant, researcher)	
Were ther any co‐interventions (Yes/no/none/noted) If yes, what were they?	
Number of missing participants and reasons for being missing?	
Cost of intervention if available (e.g. per unit, per pupil)	
Is specific equipment required to replicate the intervention? (Yes/no/not sure)	
Provide detail of the equipment required for the intervention	
Duration of follow‐up	
Was sustainability discussed? (Yes/no/unclear)	
State details of the control/comparison condition (e.g. business as usual, waiting list, another intervention, etc)	
Was an analytic framework applied? (Yes/no/unclear)	
Were all variables reported in the results section as specified in aim/rationale? (Yes/no/unclear)	
Specifc details of the primary outcome and measure (specifically the name of the measure)	
Specifc details of the secondary (if any) outcome and measure (specifically the name of the measure)	
Time points at which outcome measures were assessed	
Time points at which outcome measures were reported	
Details of results (e.g. Mean, SD, No. of participants etc) Please provide page number for results section.	
When and how was the outcome measure applied (e.g. during class time, in a quiet room on an individual basis, etc)	
Unit of measurement	
Was the outcome measure validated? (Yes/no/unclear)	
Did imputation of missing data occur?(Yes/no/unclear)	
Was there adequate power in the study? (Provide evidence)	
Is the full original data available? (Yes/no/unclear) Were the authors conclusions included in the paper?	
Were there any obvious shortcomings in the study?	
Other relevant socio‐ demographics for each group	
Specifc details on withdrawals and exclusions, specify numbers and reasons	
Did random sequence generation occur?(Yes/no/unsure)	
Support for judgement	
Did allocation concealment occur?(Yes/no/unsure)	
Support for judgement	
Did blinding of those delivering the intervention occur? (Yes/no/unsure)	
Support for judgement	
Did blinding of outcomes assessment occur? (Yes/no/unsure)	
Support for judgement	
Was there incomplete outcome data? (Yes/no/unsure)	
Support for judgement	
Was there selective outcome reporting? (Yes/no/unsure)	
Support for judgement	
Was there any evidence of any additional bias (state explicitly)	
Is the context of study adequately described? (Yes/no/unclear) Are the aims of the study clearly reported? (Yes/no/unclear)	
Is there an adequate description of the sample used in the study and how the sample was identified and recruited? (Yes/no/unclear)	
Is there an adequate description of the methods used in the study to collect data? (Yes/no/unclear)	
Is there an adequate description of the methods of data analysis? (Yes/no/unclear)	
Is the study replicable from this report? (Yes/no/unclear)	
Do the authors avoid selection bias? (Yes/no/unclear)	
Are there ethical concerns? (Yes/no)	
Is there sufficient justification for why the study was done the way it was? (Yes/no)	
Was the choice of research design appropriate for addressing the research question posed? (Yes/no)	
Have sufficient attempts been made to establish the reliablity of data analysis?(Yes/no)	
Have sufficient attempts been made to establish the validity of data analysis?(Yes/no)	
Are there any other sources of error/bias? (Yes/no)If yes, please specify	
How generalisable are the results?	
Weight of evidence in justifying study question/conclusions (e.g. high, medium or low)	
